# Mechanical Performance of Structural Polymethyl Methacrylate Joints at Different Temperatures

**DOI:** 10.3390/polym16233243

**Published:** 2024-11-22

**Authors:** Chenxing Kang, Lei Peng, Yantao Li, Jinhui Zong

**Affiliations:** 1 School of Civil Engineering and Transport, Hebei University of Technology, Tianjin 300401, China; kkccxx2001@163.com (C.K.); lytao@hebut.edu.cn (Y.L.); 2 Tianjin Teda Fire Protection Technology Co., Tianjin 300380, China; tjtdfire@163.com

**Keywords:** structural PMMA, bulk polymerization, tensile testing, mechanical properties, constitutive model

## Abstract

This paper introduces a novel technique for enhancing the joint strength in structural acrylic glass (polymethyl methacrylate, PMMA) under thermal cycling conditions. By employing bulk polymerization, the strength of PMMA joints was significantly reinforced. Tensile assessments from 20 °C to 140 °C were conducted to evaluate the mechanical properties of acrylic joints under varying temperature conditions. A constitutive model was established to correlate the strength of both the base material and the joints with temperature variations. The tensile test outcomes demonstrated that the innovative bulk polymerization method under thermal cycling conditions effectively increased the joint material strength to reach up to 90% of the base material’s strength, and the post-thermal cycling tests demonstrate that post-thermal cycling has essentially no impact on the strength and modulus. This advancement in joint strength enhancement not only expands the potential applications of acrylic glass in architectural structures but also lays a substantial theoretical foundation for construction practices.

## 1. Introduction

Plexiglas (PMMA) is a versatile polymer material known for its low density, excellent light transmission, and high strength. Owing to these superior mechanical and optical properties, PMMA has been widely used in diverse fields, such as aerospace, automotive, marine engineering, and high-energy physics. In light of recent improvements in material quality and emerging architectural trends, Plexiglas is increasingly utilized as a facade material in building exteriors [1,2,3,4].

Extensive research has been conducted on the properties of Plexiglas. Studies initially focused on the influence of temperature and strain rate on the mechanical behavior of this material. For instance, Richeton et al. [5] examined the uniaxial compression of PMMA and noted an increase in yield stress with lower temperatures and higher strain rates. Ionita et al. [6]’s work with a dynamic mechanical analyzer revealed that Plexiglas displays significant viscoelastic changes around its glass transition temperature. Wu et al. [7] discussed the deformation characteristics of PMMA at various temperatures, while Liu [8] investigated the fatigue life of U-notched PMMA plates at different temperatures, and Gao [9] studied the creep constitutive model of PMMA at different temperatures and successfully simulated its creep behavior. In addition, other scholars have conducted a series of studies on Plexiglas based on strain rate and temperature [10,11,12,13].

In aerospace applications, the focus has shifted to the performance of Plexiglas under high strain rates of unidirectional stretching. Yadav and Jaya [14] explored how the feature size and sample volume affect the damage resistance of PMMA. Zhang et al. [15] investigated the compressive behavior of PMMA when subjected to temperatures ranging from room to high temperatures, while others [16,17,18,19] used high-speed photography and digital image correlation to study its dynamic fracture behavior under impact loads, such as bird strikes.

In ocean engineering, Du et al. [20] investigated the structural impact performance of PMMA materials in underwater engineering applications, initially proposing a simplified plate model for spherical hulls subjected to concentrated impact loads. Kochanov et al. [21]’s research on truncated conical portholes suggested the use of an optimal cone angle for minimizing stress, and Di et al. [22] examined the effects of defects and aspect ratios on the load capacity of spherical PMMA shells. Wang et al. [23] performed quasistatic loading-unloading cyclic tests on PMMA windows within a high-pressure chamber, providing an experimental foundation and theoretical basis for the design specifications of manned submersible observation windows.

In the construction industry, the emphasis is on the structural behavior of Plexiglas under various conditions. Wang et al. [24] investigated the crack resistance of Plexiglas panels, and Huang et al. [25] used a scale-down model to study the load-bearing capabilities of Plexiglas in the future garden of Jiangsu Garden Expo Park and conducted both long-term and short-term tests.

Given the diverse applications of PMMA across different fields, the material properties and focal points of interest vary. Structural PMMA emphasizes tensile properties, particularly the mechanical performance at the joints. It remains to be experimentally verified whether conclusions from other fields can be directly applied to structural PMMA. Consequently, this paper reports tensile tests conducted to investigate the mechanical properties of bulk-polymerized structural PMMA at elevated temperatures.

## 2. Experimental Design

### 2.1. Specimen Design

In construction applications, Plexiglas panels often need to be considerably thick, ranging from tens to even hundreds of millimeters. Following the guidelines of the ASTM D638 [26] standard, panels thicker than 14 mm should be machined down to 14 mm for testing purposes. 

**Figure 1:**
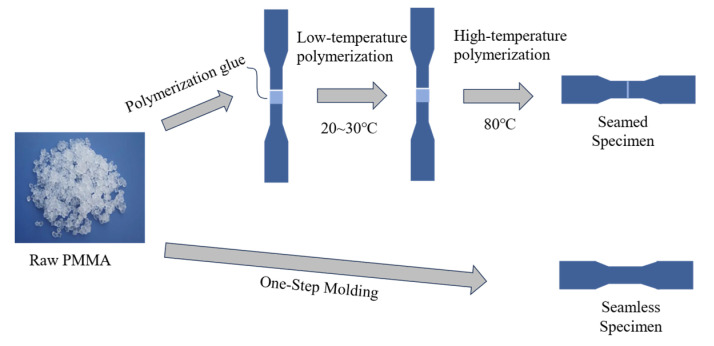
Specimen manufacturing processes.

**Figure 2:**
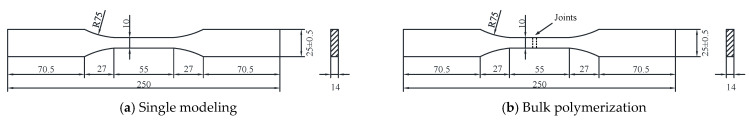
PMMA specimens.

A novel acrylic-bonding technique applied in construction involves specimens undergoing bulk polymerization at 20–30 °C, followed by heat treatment at 80 °C and subsequent cooling to yield the final product. To investigate the strength at the joints of specimens fabricated through this new technique, comparative experiments were designed between jointed specimens produced by bulk polymerization and those created through a single-molding process, with the splice in each specimen located centrally. We used PMMA samples from Tomson New Material Technology Co. (China, Jiangsu). Figure 1 shows the specimen fabrication process, while Figure 2 illustrates the dimensions of the Plexiglas specimens prepared for the experimental analysis.

### 2.2. Methodology

#### 2.2.1. PMMA Tensile Tests at Various Temperatures

**Figure 3:**
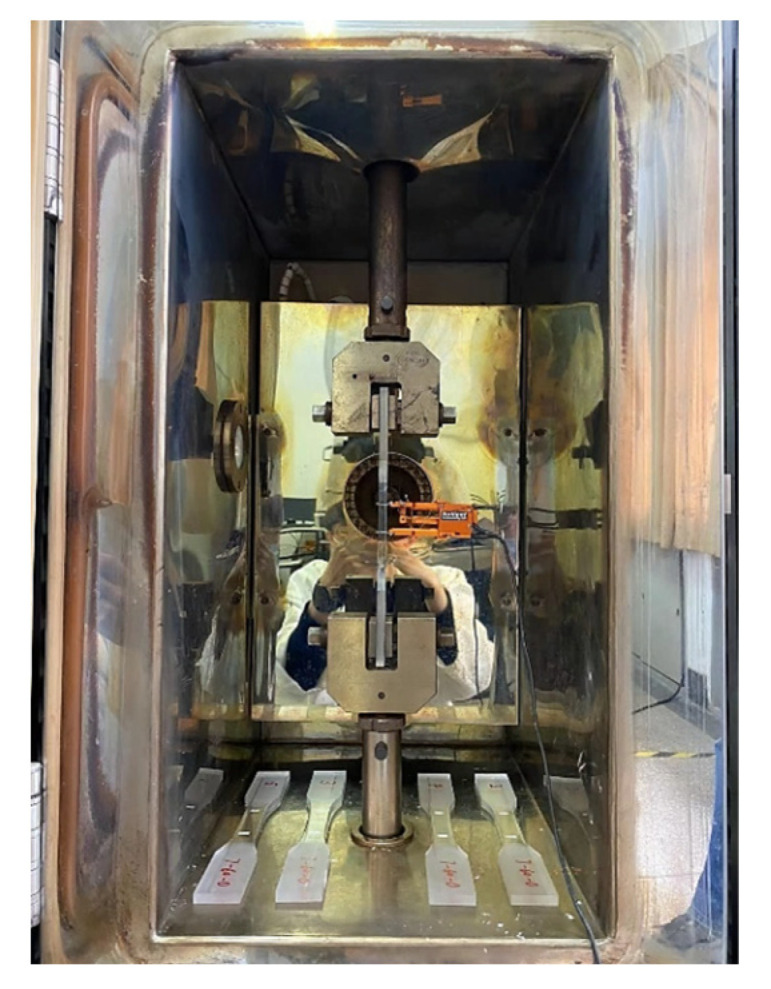
High-temperature tensile test setup.

The experimental methodology adhered to GB/T 1040.2-2022 “Determination of Tensile Properties of Plastics” [27]. Uniaxial tensile tests were conducted using an electronic tensile testing machine. High-temperature tensile tests were carried out inside a numerically controlled high-temperature chamber. Initially, the specimens were preheated in the chamber until the temperature stabilized. After maintaining the set temperature for 30 min, tensile tests were performed within the high-temperature environment. This process is graphically depicted in Figure 3.

The specimens were subjected to heating at temperatures of 20 °C, 40 °C, 60 °C, 80 °C, 100 °C, 120 °C, and 140 °C. To ensure the validity and reliability of the test results, each specimen type was tested five times at each temperature level. The tensile tests were executed at a uniform rate of 10 mm/min. Details of the experimental protocol are outlined in Table 1.

#### 2.2.2. Post-Thermal Cycling PMMA Tensile Tests

One experimental protocol was designed to evaluate the mechanical property alterations of structural PMMA following exposure to fire in engineering applications. The specimens without seams, as depicted in Figure 2b, were subjected to heating followed by cooling to 20 °C prior to uniaxial tensile testing. The heating temperatures were set at 40 °C, 60 °C, 80 °C, 100 °C, 120 °C, and 140 °C, with quintuplicate experiments conducted at each temperature to ensure accuracy. The experimental conditions are detailed in Table 2.

## 3. Results and Analysis

### 3.1. Tensile Test at Various Temperatures

#### 3.1.1. Forms of Failure

**Figure 4:**
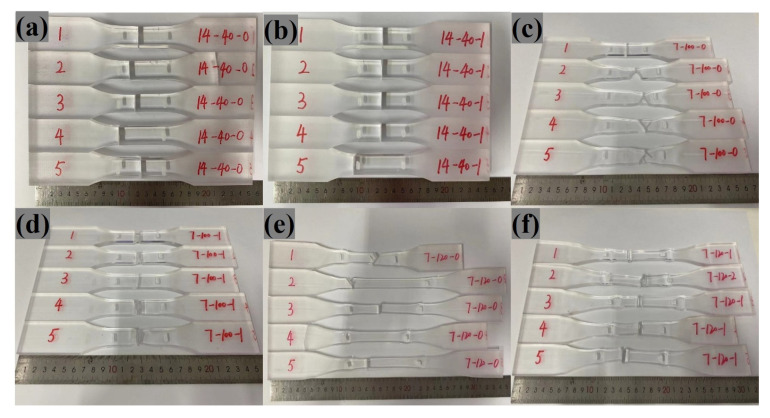
Modes of specimen failure (temperature, number of splices): (**a**) 40 ℃, 0; (**b**) 40 ℃, 1; (**c**) 100 ℃, 0; (**d**) 100 ℃, 1; (**e**) 120 ℃, 0; and (**f**) 120 ℃, 1.

In the present experiment, the specimens demonstrated a variety of failure modes at different testing temperatures (refer to Figure 4). These modes can be broadly categorized into three distinct types, outlined below.

Initially, within the lower temperature range of 20 °C to 60 °C, the specimens exhibited rapid failure without significant deformation, characteristic of brittle fractures. As temperatures approached the glass transition zone (80 °C to 100 °C), the ductility of the specimens markedly increased. Under sustained loading, the specimens gradually elongated and necking occurred, indicative of plastic deformation, transitioning the mode of failure to ductile fracture. Finally, surpassing the glass transition temperature range of 120 °C to 140 °C, the specimens’ capacity for deformation was further enhanced, exhibiting pronounced plasticity while maintaining ductile failure characteristics. The experimental findings reveal a close correlation between the mode of failure and the testing temperature. With increasing temperatures, the material undergoes a shift from brittle to ductile failure, which, when reflected in PMMA structures like panels and beams, results in increased deflections, compromising their functionality. Despite this, the ductile nature of the failure prolongs the window for evacuation and emergency response.

Additionally, Figure 4 demonstrates that the failure points of seamed specimens are always at the seams, regardless of the temperature range, while the failure of seamless specimens occurs at stress concentrations caused by geometric changes, suggesting that the strength recorded for seamed samples is indicative of the seam strength.

#### 3.1.2. Stress–Strain Curves

In uniaxial tensile or compression experiments, the data typically collected are presented as nominal stresses and nominal strains, which correspond to engineering stresses and strains, respectively. These values are calculated using the following formulas:

(1) $$\sigma_{nom}\;=\;\frac{F}{A_{0}}\varepsilon_{nom}\;=\;\frac{\Delta l}{l_{0}}$$

where $\;\sigma_{\mathrm{nom}}$ is the engineering stress (MPa); F is the tensile load (N); $A_{0}$ is the initial cross-sectional area of the specimen (mm^2^)$;\;\varepsilon_{\mathrm{nom}}$ is the engineering strain; Δl is the displacement (mm); and $l_{0}$ is the initial length of the specimen (mm).

**Figure 5:**
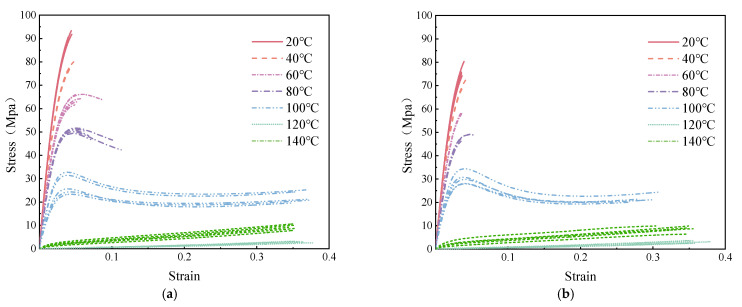
Stress-strain curves at various temperatures: (**a**) seamless specimen and (**b**) seamed specimen.

During the process through which a large deformation occurs, significant changes in the cross-sectional area of a material occur, rendering nominal stress–strain measurements insufficient for accurately capturing the material’s behavior. To precisely describe these changes, it is necessary to employ true stress and true strain (also referred to as logarithmic strain). The conversion formulas that relate true stress and strain to nominal stress and strain are as follows:

(2) $$\sigma_{true}\;=\;\frac{F}{A}\;=\;\frac{F}{A_{0}\frac{l_{0}}{l}}\;=\;\sigma_{nom}(1\;+\;\varepsilon_{nom})$$

(3) $$\varepsilon_{true}\;=\;\int_{l_{0}}^{l}\frac{dl}{l}\;=\;\ln(\frac{l}{l_{0}})\;=\;\ln(1\;+\;\varepsilon_{nom})$$

where $\sigma_{\mathrm{true}}$ is the true stress (MPa); l is the instantaneous length of the specimen (mm); and A is the instantaneous cross-sectional area of the specimen (mm^2^). Utilizing the aforementioned methodology, the processed data from the tensile tests at varying temperatures are presented in Figure 5. When referring to stress–strain data, it is assumed that the data have been converted using the appropriate formula, and, unless otherwise specified, the data provided represent true stress and true strain values.

Within the lower thermal regime (20 °C to 60 °C), the specimens exhibit characteristics akin to those of vitreous materials, with significant elastic modulus and ultimate tensile strength values. The corresponding stress-strain profiles lack an identifiable yield point, suggesting that deformation remains within the elastic domain. The fracture strain is confined to a range of 3% to 7%, culminating in brittle fracturing. Upon transitioning to the glass transition zone (80 °C to 100 °C), a pronounced shift to plastic deformation is observed. The material exhibits yield behavior, accompanied by strain-softening and necking phenomena. It is worth noting that a plateau in the stress–strain response of the specimen occurs at 100 °C. This mechanical behavior is interpretable through the lens of polymer physics. Plexiglas, which is composed of polymeric groups and macromolecular chains, undergoes structural modifications upon the application of a load. These modifications manifest as translational and rotational movements of the chains and their side groups, resulting in strain softening. Simultaneously, these molecular rearrangements induce a transition from a disordered to an ordered state among the molecules, enhancing the resistance to entropic forces and leading to strain hardening after cold drawing, ultimately yielding ductile failure. In scenarios where the temperature exceeds the glass transition threshold (120 °C to 140 °C), the specimens demonstrate behavior analogous to that of highly elastic, rubber-like materials. The stress-strain relationship under these conditions is approximately linear, and a marked decrease in both the strength and elastic modulus of the material is observed.

### 3.2. Thermal Cycling Test

**Figure 6:**
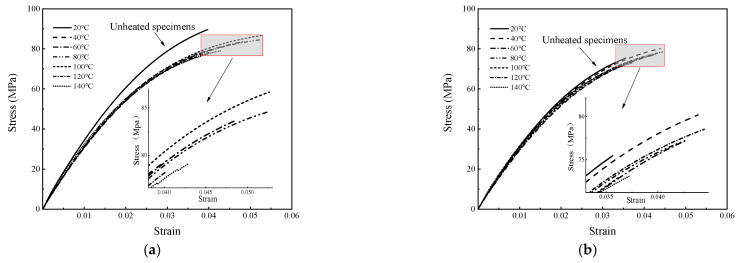
Stress-strain curves after thermal cycling: (**a**) seamless specimen and (**b**) seamed specimen.

Quasistatic uniaxial tensile tests were conducted in accordance with the experimental protocols detailed in Table 2. From the test, we derived the true stress-strain curves for each specimen. Figure 6 demonstrates the effect of different degrees of heating on the material stress–strain curve under thermal cycling conditions. The tensile attributes of the spliced specimens after thermal cycling exhibited a decrease in strength compared with those of the pristine, unheated controls. Concurrently, an increase in ductility was discernible. This phenomenon is ostensibly akin to the metallurgical tempering process, wherein reheating ostensibly serves to ameliorate the residual stresses imparted during a specimen’s genesis, thereby attenuating a material’s inherent brittleness.

Moreover, it is imperative to note that the mechanical properties of the specimens subjected to a heating apex of 140 °C are the most suboptimal within the cohort. However, an examination of the stress-strain trajectories after thermal intervention revealed a nonmonotonic relationship with respect to the heating temperature gradient. The confluence of these curves, characterized by a narrowed dispersion, indicates that the zenith of thermal exposure does not exert a pronounced influence on the mechanical characteristics of the specimens in the post-fire phase.

Therefore, when heated to 140 °C and then cooled in the thermal cycle, the decrease in material properties was greater than at other temperatures, but there was no significant difference, and, in general, the mechanical properties of the material were essentially unchanged after the fire.

## 4. Quantitative Analysis of Factorial Impacts on Mechanical Properties

### 4.1. Effects of Temperature Variations

**Figure 7:**
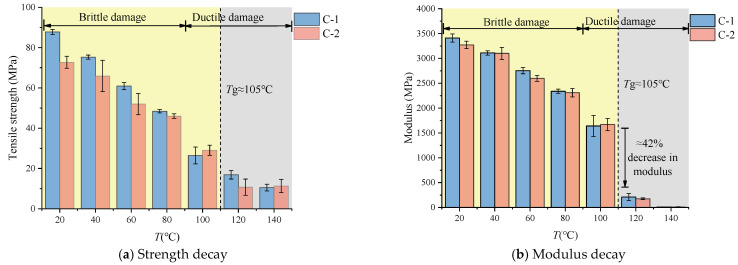
Mechanical properties affected by temperature.

The collation of experimental data yields Figure 7, which reveals significant variability in the strength of specimens with seams, attributable to the limited ductility at the joint. As the temperature increases, the ultimate load-bearing capacity of the specimens diminishes, with the seam strength marginally lower than that of the base material, although the difference is not substantial. At temperatures of 100 °C and 140 °C, the seam strength even surpasses that of the base material. This may be due to the mechanical properties of the material becoming more variable after surpassing the glass transition temperature (Tg), but, overall, the strength at the seam remains consistently above 85% that of the base material, meeting the requirements for engineering applications.

Figure 7b presents a comparison of material moduli, showing a trend in the elastic modulus which is similar to that of the strength. Notably, a significant decline in the elastic modulus of both materials occurs after the glass transition temperature (Tg), with a reduction of up to 42%. Based on this observation, the subsequent research on the material’s constitutive behavior is focused on the range before Tg (20~100 °C).

### 4.2. Effects of Cooling on Heated Sheets

**Figure 8:**
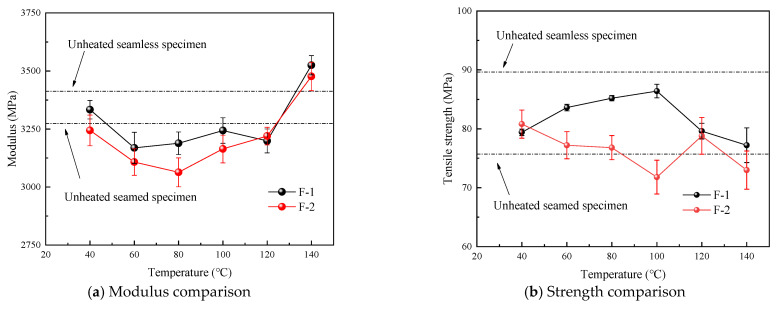
Modulus and strength variation.

Figure 8a illustrates the results obtained from analyzing the high-temperature postcooling test data. The figure highlights the impact of thermal cycling on the elastic moduli of two distinct Plexiglas specimens—one with splices and one without splices. The elastic moduli of both specimens decreased within the temperature range of 40 °C to 120 °C. Specifically, the elastic modulus of the seamless specimen decreased to 92~96% of its original value prior to thermal exposure, while that of the spliced specimen decreased to 94–97% of its initial modulus. Notably, at a preheating temperature of 140 °C, an increase in the elastic modulus was recorded that surpassed that of the pristine, unheated specimen. Figure 8b reveals that thermal cycling differentially affects the tensile strengths of the two types. The unspliced specimen showed a decrease in tensile strength to 79.2–87.6% of the baseline value. In contrast, the strength of the spliced specimen after thermal cycling was symmetrically comparable to the tensile strength of the unmodified material.

Overall, thermal cycling results in a slight reduction in the strength of the PMMA base material, while having no detrimental effect on the seam strength. It can be inferred that the continued use of PMMA post fire exposure has a negligible impact on structural safety in engineering applications.

## 5. Establishment of Constitutive Modeling

It is well established that polymers undergo a significant transition in mechanical properties upon heating beyond the glass transition temperature (Tg). In engineering applications, it is generally accepted that structural PMMA loses its load-bearing capacity beyond its Tg of 105 °C. Consequently, the temperature range under investigation in the following study is confined to 20–100 °C.

### 5.1. Matsuoka Model Development and Validation

Matsuoka introduced a phenomenological constitutive model for glassy polymers, represented by the following equation:

(4) $$\sigma (T,\varepsilon )\;=\;E_{k}\;\times \;\varepsilon \;\times \;\exp\left(k_{1}\varepsilon \right)\;\times \;\exp\left(k_{2}\varepsilon^{k_{3}}\right)$$

The formula encompasses four parameters, which equip the model with the ability to describe complex mechanical characteristics such as pre-yield non-linear elastic behavior, the yielding process, strain softening, and strain hardening.

**Figure 9:**
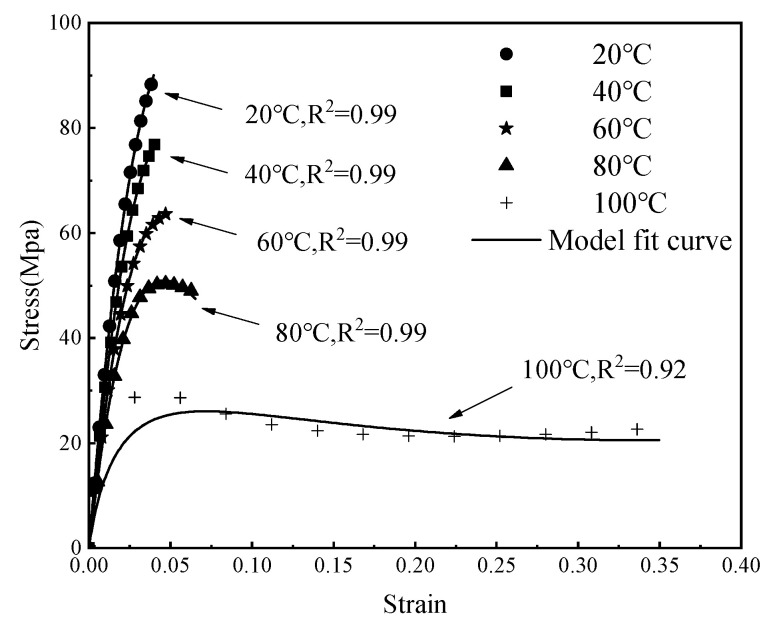
Model fitting effectiveness.

Employing the average experimental data from Condition C-1 (20–100 °C), the new model proposed in this article was applied to fit and validate the experimental data. The fitting results, illustrated in the accompanying Figure 9, indicate a commendable accuracy of the model. Subsequently, all experimental data were fitted using this model, and the fitting parameters for the entire dataset are detailed in Table 3.

### 5.2. Model Improvement and Validation

**Figure 10:**
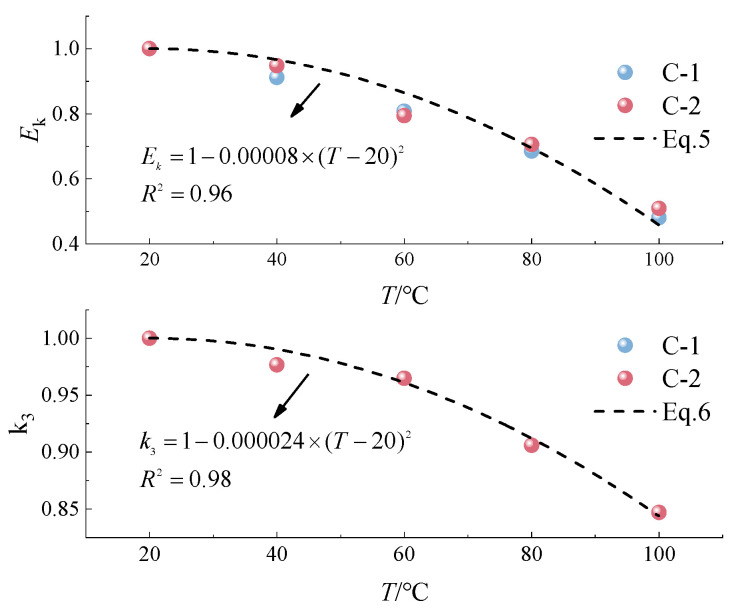
Temperature model fit.

Analyzing the dataset in Table 3, it can be observed that the values of $E_{k}$ and $k_{3}$ decrease with increasing temperatures. To enhance the universality of the model, the values of $E_{k}$ and $k_{3}$ at various temperatures were normalized, revealing a quadratic relationship in their decline. Consequently, this study introduces temperature-dependent decay models for $E_{k}$ and $k_{3}$, detailed in Equations (5) and (6), respectively. The model’s efficacy in capturing the trend in parameter changes is well illustrated in Figure 10.

(5) $$E_{k}\;=\;E_{20}*(1\;-\;0.00008*{\left(T\;-\;20\right)}^{2})$$

(6) $$k_{3}\;=\;{k_{3}}^{20}*(1\;-\;0.000024*{\left(T\;-\;20\right)}^{2})$$

where $E_{k}$ is the modulus at a given temperature (GPa); $E_{20}$ is the baseline modulus at 20 °C (GPa); $k_{3}$ is the temperature-dependent exponential parameter; ${k_{3}}^{20}$ is the parameter value at 20 °C (same value for the base material and the splicing parameter, 0.85); and T is the heating temperature (°C).

Based on the conclusions and data mentioned above, a constitutive model for PMMA stress as a function of strain and temperature can be established. The fitting data reveal that the three parameters determining the shape of the curve fluctuate within a certain range. The average values of these three parameters are taken as the shape parameters of the model curve. The values of $E_{k}$ and $k_{3}$ change with the temperature and based on the quadratic relationship derived from Equations (5) and (6). Hence, the model proposed in this paper is as presented in Equation (7):

(7) $$\begin{array}{c} \left\{\begin{array}{l} \sigma (T,\varepsilon )\;=\;E_{k}(T)\;\times \;\varepsilon \;\times \;\exp\left(14.46\;\times \;\varepsilon \right)\;\times \;\exp\left(-18.11\;\times \;\varepsilon^{k_{3}(T)}\right)\\ E_{ko}(T)\;=\;3.81\;\times \;\left[1\;-\;8e^{-5}\;\times \;{\left(T\;-\;20\right)}^{2}\right]\mathrm{for}\;\mathrm{base}\;\mathrm{material}\;\\ E_{kj}(T)\;=\;3.68\;\times \;\left[1\;-\;8e^{-5}\;\times \;{\left(T\;-\;20\right)}^{2}\right]\mathrm{for}\;\mathrm{joints}\\ k_{3}(T)\;=\;0.85\;\times \;\left[1\;-\;2.4e^{-5}\;\times \;{\left(T\;-\;20\right)}^{2}\right] \end{array}\right. \end{array}\left(20\;{}^{\circ}C\;\leq \;T\;\leq \;100\;{}^{\circ}C\right)$$

where $E_{ko}$ is the base material’s modulus at a given temperature (GPa); $E_{kj}$ is the joint’s modulus at a given temperature (GPa); $k_{3}$ is the temperature-dependent exponential parameter; and T is the heating temperature (°C).

**Figure 11:**
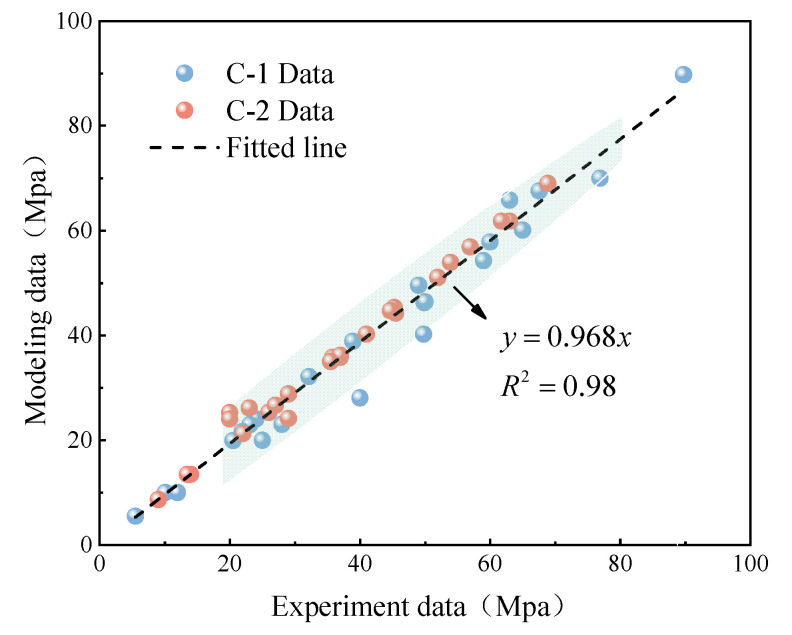
Analysis of model reliability.

The stress–strain curves calculated using the model proposed in this paper were uniformly sampled at five points for each temperature level to compare with the experimental data. The comparison is depicted in Figure 11, where the correlation line between model data and experimental data has a slope of 0.968, and the coefficient of determination R^2^ is 0.94. This indicates that the model can accurately describe the mechanical behavior of PMMA, providing a theoretical foundation for subsequent finite element analysis.

## 6. Conclusions

This paper presents a novel technique aimed at bolstering the joint strength of structural PMMA when subjected to thermal cycling. Through meticulous tensile testing across various temperatures, we evaluated the mechanical integrity of the acrylic joints. Findings indicate that joints created through bulk polymerization maintain robust mechanical properties, with strengths surpassing 85% of the base PMMA material and a consistent elastic modulus, albeit with a minor decrease in the fracture strain at the joints. Our research corroborates existing studies that document a reduction in both the strength and elastic modulus of structural acrylic glass with rising temperatures, reinforcing the need for their consideration in the development of future PMMA constitutive models. Utilizing the Matsuoka model, we have formulated a new constitutive model that precisely forecasts the interplay between temperature and strength, thereby broadening the scope of PMMA applications in engineering. However, the slight strength decrease observed in PMMA during thermal cycling, which does not affect seam integrity, should be interpreted with caution due to experimental limitations, including the scale of fire simulation and the exposure duration. It is crucial for subsequent studies to confirm these preliminary results in more varied conditions and delve into the endurance mechanisms that preserve the spliced Plexiglas specimens’ robustness.

## Figures and Tables

**Table 1:** Tensile testing of PMMA at various temperatures.

Group	Specimen Thickness (mm)	Splicing of Specimens	Test Temperature (°C)	Strain Rate (mm/min)
C-1	14	Single modeling (seamless)	20, 40, 60 80, 100 120, 140	10
C-2	14	Bulk polymerization (seamed)		

**Table 2:** PMMA stretching protocol post thermal cycling.

Group	Specimen Thickness (mm)	Splicing of Specimens	Heating Temperature (°C)	Strain Rate (mm/min)
F-1	14	Single modeling (seamless)	40, 60, 80 100, 120, 140	10
F-2	14	Bulk polymerization (seamed)		

**Table 3:** Model parameters.

Group	*T* (°C)	$E_{k}$ (Gpa)	$k_{1}$	$k_{2}$	$k_{3}$
C-1	20 °C	3.81	14.05	−25.18	0.85
	40 °C	3.39	17.34	−12.06	0.83
	60 °C	2.95	10.02	−14.87	0.82
	80 °C	2.79	21.07	−15.69	0.77
	100 °C	2.31	14.45	−18.12	0.72
C-2	20 °C	3.68	8.11	−21.18	0.85
	40 °C	3.15	12.44	−15.56	0.83
	60 °C	2.87	11.36	−17.52	0.82
	80 °C	2.74	21.35	−22.86	0.77
	100 °C	2.37	14.37	−18.06	0.72
Average	——		14.46	−18.11	——

Note: $E_{k}$ is the modulus at a given temperature (GPa); $k_{1}$, $k_{2}$, and $k_{3}$ are the temperature-dependent exponential parameters.

## Data Availability

Data are contained within this article.
